# A Straightforward Approach to Create Ag/SWCNT Composites

**DOI:** 10.3390/ma14081956

**Published:** 2021-04-14

**Authors:** Monika Rdest, Dawid Janas

**Affiliations:** 1 Department of Materials Science and Metallurgy, University of Cambridge, 27 Charles Babbage Rd, Cambridge CB3 0FS, UK; Monika.Rdest@gmail.com; 2 Department of Organic Chemistry, Bioorganic Chemistry and Biotechnology, Silesian University of Technology, B. Krzywoustego 4, 44-100 Gliwice, Poland

**Keywords:** silver, single-walled carbon nanotubes, composites

## Abstract

Flexible and conductive materials have a high application potential across many parts of modern life. In this work, thin free-standing films from single-walled carbon nanotubes (SWCNTs) were doped with Ag to enhance their electrical conductivity. A facile method to integrate these two materials is described herein. As a consequence, the material exhibited a six-fold boost to the electrical conductivity: an increase from 250 ± 11 S/cm to 1721 ± 125 S/cm. Interestingly, the specific conductivity remained at a comparable level upon doping, so the material was deemed promising in exploitation fields whereweight is of the essence. Furthermore, the material showed good bending characteristics, thereby revealing its applicability in flexible electronics.

## 1. Introduction

The development of civilization puts more and more burden on materials. Unfortunately, today’s progressive digitization and gadgetization seem to outpace the increase in the capabilities of the classical materials that have been serving the society for many years [1]. For instance, copper or aluminum, which are at the heart of power engineering [2], rapidly approach their performance limits [3]. Similarly, the technological constraints of silicon make further advances in microelectronics challenging [4]. The discovery of nanomaterials emerged as a potential solution to this burning problem.

One of the most notable and versatile nanomaterials classes, ever since they were first observed, is those made of carbon. Carbon nanotubes (CNTs) [5,6] and graphene [7], popularized at the turn of the 20th and 21st century, have already revealed a set of peculiar and very promising properties. Leading electrical [8,9,10], thermal [11,12,13], and mechanical [14,15,16] performance puts them at the forefront of research. They are envisioned as possible successors to typical metals prone to corrosion [17]. Moreover, the consequences of their mining are a growing environmental concern [18,19,20]. Their acquisition is restricted to specific locations, which is susceptible to geopolitical complications.

To ease the transition between metals and nanomaterials, the research community investigates the possibility of interfacing these two together. Such a combination also often results in synergy where the composite material offers a new level of performance or functionalities. For instance, the fusion of copper and nanocarbon already results in tangible benefits [3,21,22,23,24]. When such species were joined by powder metallurgy [25,26], electroplating [27,28], or electroless deposition [29,30], substantial improvements to the properties of neat Cu were observed. The reports show that the characteristics of Cu such as electrical (current carrying capacity), mechanical (hardness, tensile strength), and thermal (thermal conductivity, temperature coefficient of resistance, coefficient of thermal expansion) can be readily enhanced this way. Many more metal species can be integrated with nanocarbon to give favorable outcomes. For instance, such a combination can produce sensitive electrochemical sensors for detection of various analytes [31,32,33].

A metal, which is a particularly good candidate for such a procedure is silver, which can be used to map heterogeneity of electrical properties of carbon structures [34,35,36]. Importantly, Ag has the highest electrical conductivity among all metals at room temperature, which amounts to 6.30 × 10^7^ S/m [37]. It is also ductile and malleable. However, to reach the level of flexibility required by modern electronics, silver commonly needs to be nanostructured to take the shape of e.g., silver nanowires (AgNWs) [38,39,40]. This complication makes the application of silver less competitive, despite its merits, since a rather expensive starting material has to undergo additional processing steps. Recently, Lekawa-Raus et al. observed that the addition of commercially available silver nanoparticles (AgNPs) to multi-walled CNT fibers improves the electrical conductivity of the network by reducing the contact resistance [41]. Up to 80% reduction in electrical resistance was observed (0.018 S/cm) upon incorporating a small amount of AgNPs, which appeared in the SEM micrographs as nanoclusters distributed well on the surface. Ma and co-workers also combined AgNPs with MWCNTs, which gave rise to the six-fold enhancement of electrical conductivity of the material from 5.15 to 30.53 S/cm [42]. Next, Namura and colleagues demonstrated that upon addition of AgNPs to single-walled carbon nanotube (SWCNT) films the electrical conductivity can be increased from 37.8 to 50.6 S/cm after annealing at 723 K under a nitrogen atmosphere [43]. Since these values are far from the electrical conductivity of pure Ag, there is definitely room to improve the performance of such nanocomposites.

In this work, SWCNT networks were heavily loaded with silver to investigate what is the highest possible enhancement of their electrical conductivity. Conductive silver paint was added to the SWCNT dispersion, from which a composite Ag/SWCNT film was directly formed. The inclusion of Ag in situ enabled homogeneous distribution of metal throughout the whole volume of the material. This resulted in the production of highly conductive composites while preserving the appreciable flexibility of the material.

## 2. Materials and Methods

### 2.1. Compounds and Materials

Large-diameter SWCNTs (Tuball™; OCSiAl, Leudelange, Luxembourg), Ag conductive paint (SCP03B; Electrolube, Ashby-de-la-Zouch, UK), acetone (Avantor, Gliwice, Poland), and toluene (Avantor, Gliwice, Poland) were procured from the indicated parties. All of the chemicals had a p.a. class, so they were employed without exercising any purification steps. According to the information from the manufacturer, Ag conductive paint contains 45% of Ag by weight. The remainder is a matrix of low boiling point solvents to enable their facile evaporation upon the application i.e., 1-ethoxypropan-2-ol, ethanol, acetone, and ethyl acetate in the descending order of weight.

### 2.2. Manufacture of Free-Standing SWCNT-Based Films

**Figure 1:**
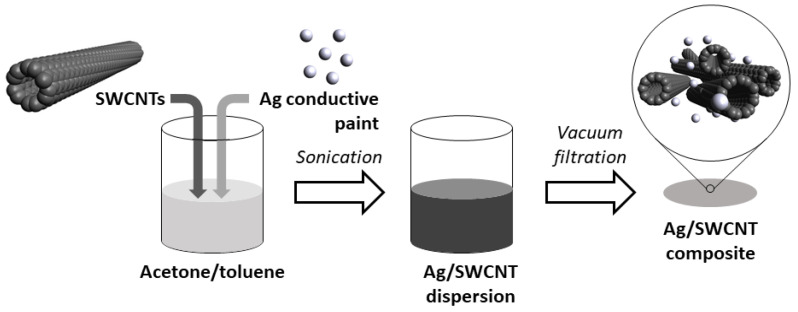
Manufacture of Ag-SWCNT (single-walled carbon nanotube) composites in the form of free-standing films by vacuum filtration. The microstructure of the material was modeled by Avogadro: an open-source molecular builder and visualization tool [45].

SWCNT films were prepared according to a technique reported by us previously [44] (Figure 1). The exception is that, for this study, Ag particles were additionally incorporated into the material to enhance its electrical conductivity. In summary, 150 mg of dry SWCNTs stored in a desiccator were added to 79 mL of a mixture of acetone and toluene (1:1 by weight). Next, 1 mL of Ag conductive paint was introduced either from the parent concentration or diluted ten-fold with acetone and toluene mixture (1:1 by weight). The former and latter formulations are in this work referred to as Ag-SWCNTs (high) and Ag-SWCNTs (low). SWCNTs, Ag, and solvents were then ultrasonicated at 100% amplitude (UP200St sonicator; Hielscher, Teltow, Germany) over ice to reach a uniform dispersion. A total of 10 min of processing was required in each case. The produced dispersions were vacuum filtered through the PTFE membrane filters (diameter: 47 mm, pore size: 0.45 µm; Fisherbrand, Ottawa, ON, Canada). The low affinity of SWCNTs to PTFE enabled straightforward delamination of the material from the substrate to give free-standing films for the study.

To measure their electrical conductivity, 3 mm × 20 mm specimens were cut out from the circular SWCNT membranes obtained after filtration.

Neat SWCNT films were manufactured as a reference for this study as well. In that case, the process of film formation was conducted analogously, as highlighted above, but it was carried out in the absence of Ag conductive paint. To keep the processing volume consistent, SWCNTs were added to 80 mL of a mixture of acetone and toluene (1:1 by weight).

### 2.3. Characterization

Raman spectroscopy (inVia Renishaw system, λ = 633 nm, Wotton-under-Edge, UK) was used to determine the crystal perfection of the employed SWCNTs. The spectra were measured from 0 to 3500 cm^−1^. To minimize the possible influence of material inhomogeneity and background noise, the spectra were acquired in multiple areas of the parent SWCNT powder and after several accumulations, respectively. Laser power was minimized (down to 0.01% of total beam intensity) to avoid possible sample overheating caused by photon absorption [32]. The average value of I_D_/I_G_ ratio was presented along with the calculated standard deviation.

Scanning Electron Microscope (SEM, FEI Quanta 250 FEG, Hillsboro, OR, USA) was used to study the microstructure of the films. An acceleration voltage of 15 kV was used in the experiment. Due to the high electrical conductivity of the specimens, there was no need to sputter them with metal. A conjugated Energy Dispersive X-ray (EDX) detector measured the chemical composition of the material from 0 to 15 keV.

A four-probe method was applied to gauge the electrical conductivity of the SWCNT films. For this purpose, 3 mm × 20 mm specimens were cut out from the parent SWCNT films and then mounted onto custom-designed sample holders. These consisted of four Cu tape terminals to create current-carrying and voltage-sensing pairs. Ag conductive paint was added to the Cu-SWCNT interface to ensure appropriate mechanical and electrical contact. A source meter (Keithley 2450 SourceMeter, Cleveland, OH, USA) was used for the experiments.

Obtained values of conductance were recalculated to conductivity by considering the dimensions of the characterized samples. The thickness of the films was determined with a micrometer screw gauge (Electronic Universal IP54, Linear Tools, Dunstable, UK). Multiple samples were analyzed to guarantee the statistical significance of the discussed data.

Weight change upon the incorporation of Ag was measured with an analytical balance (TA164i; VWR, Radnor, PA, USA) having the readability of 0.1 mg. The recorded weights were used to establish the impact of Ag addition on the specific conductivity of the material. The values of the electrical conductivity of the specimens were divided by the corresponding weights.

To demonstrate the flexibility and durability of the created Ag/SWCNT hybrids, a selected sample was bent 100 times (90 degrees bend, 1 cm bend radius). SWCNT film heavily-doped with Ag was chosen for this part, as it should be most prone to mechanical deformation. The electrical conductivity of such material—Ag/SWCNTs (high)—was measured during the execution of the bending routine.

## 3. Results

**Figure 2:**
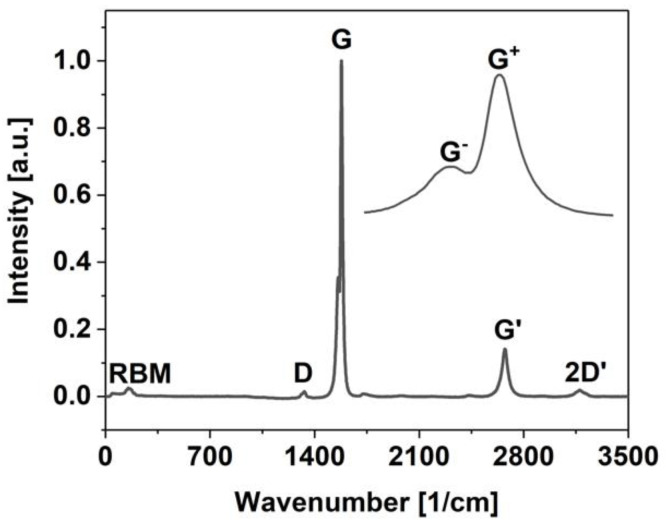
Raman spectrum of the SWCNT parent material utilized to make the free-standing films in this study. Splitting of the G band into G^−^ and G^+^ components is visualized in the inset.

**Figure 3:**
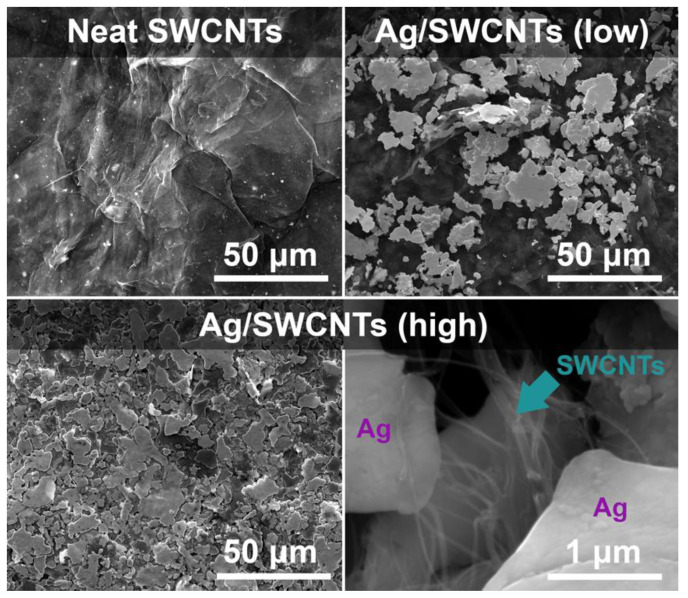
SEM micrographs of neat SWCNT films and upon doping with low (ten-fold dilution of the Ag conductive paint) and high amount of Ag (no dilution of the conductive paint).

The study was initiated with the characterization of the purity of the starting SWCNT material used to make the conductive networks by Raman spectroscopy (Figure 2). Calculation of an I_D_/I_G_ ratio enables one to probe for imperfections of the material in the form of lattice defects or functional groups. The presence of such factors is manifested by the emergence of a D-band near 1330 cm^−1^. Conversely, the G-band corresponds to the vibration of graphitic carbon atoms of sp^2^ hybridization, which is detected in the vicinity of 1580 cm^−1^ [46]. In this case, the I_D_/I_G_ ratio amounted to only 0.014, proving that the material is of very high quality. Moreover, the appearance of the Radial-Breathing Mode (RBM) and splitting of the G band into G^−^ and G^+^ components indicated that the material was indeed single-walled [47]. All these findings support the hypothesis that the films made from such material should have high electrical conductivity. We moved on to the characterization of the microstructure, which is shown in Figure 3.

The unmodified SWCNT films revealed a typical topography of networks prepared by filtration. Individual SWCNTs and their bundles constituting the film were not aligned, so the material was isotropic. The voids separating them, typically present in such a material, are expected to provide mesoporous character to the SWCNT ensemble [48,49].

A considerable difference is observed when Ag is combined with SWCNTs at the stage of the films’ formation. When a low metal content is employed—Ag/SWCNTs (low)—large scattered Ag flakes are detected. The size of the flakes ranges from a few to tens of microns in width. At the ten-fold dilution of the Ag conductive paint, the amount of added metal is insufficient to create a continuous Ag layer. Conversely, the addition of the concentrated solution of Ag conductive paint caused even coverage of the surface of SWCNTs with metal: Ag/SWCNTs (high). Nearly all the SWCNTs are coated with Ag, and it is challenging to find a location with visible SWCNTs. An example of such an area is presented in the bottom right part of Figure 3 (indicated with an arrow), wherein SWCNTs protrude from the cavity in the Ag covering. Overall, at the high concentration of Ag, the micrographs show excellent homogenization of Ag within the SWCNT matrix.

**Figure 4:**
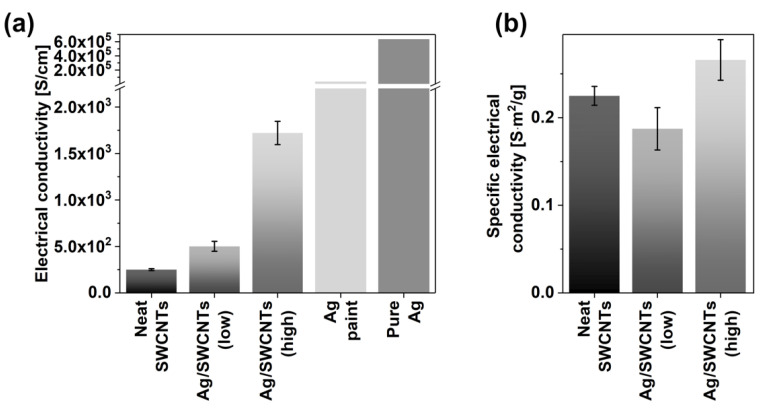
(**a**) Electrical conductivity of the SWCNT films before and after doping with a low and high amount of Ag conductive paint. The conductivity of Ag conducitve paint and pure Ag are taken from the literature and given as a reference [37,51]. (**b**) Specific electrical conductivity of the SWCNT films before and after doping with a low and high amount of Ag conductive paint.

Next, we characterized the electrical conductivity of neat SWCNTs and the composites with Ag (Figure 4). The undoped SWCNT films demonstrated the electrical conductivity of 250 ± 11 S/cm, which stayed in accordance with our previous results [50]. However, a colossal increase in this property was observed when Ag was interfaced with SWCNT films. Upon incorporating a low amount of Ag, the electrical conductivity of the network increased up to 501 ± 54 S/cm, which is a 100% improvement. Even more, when the SWCNT network was heavily loaded with Ag, the electrical conductivity rose to 1721 ± 125 S/cm. This is a boost by as much as 588%. Although this value is still far from the electrical conductivity of pure Ag equal to 6.30 × 10^5^ S/cm [37], or the Ag conductive paint iself reaching 3.33 × 10^4^ S/cm [51], one needs to note that the composite contains a plethora of grain boundaries, which disrupt the charge propagation. Nevertheless, when put in the context of other metal-SWCNT composites, the obtained results are auspicious.

Because of the lightweight of SWCNT ensembles, their ability to transport charge is often reported in terms of specific conductivity, taking into account their weight. We wanted to evaluate how the addition of Ag affects this parameter (Figure 4b). Neat SWCNT films revealed the specific conductivity of 0.23 ± 0.01 S∙m^2^/g, which is appreciable [52]. This results from the application of SWCNTs of high purity having good contact between the single SWCNTs in the network, as demonstrated by SEM imaging.

When a low and high amount of Ag was added to the material, the specific electrical conductivity was 0.19 ± 0.01 and 0.27 ± 0.02 S∙m^2^/g, respectively. This corresponded to Ag:SWCNT ratios of ca. 1:5 and 1.33:1 by weight, respectively, which was close to the ratios measured by EDX (Supplementary Materials Figure S1). Relatively large standard deviation bars result from difficulties in the determination of thickness of doped SWCNT networks. Although Ag is a heavy element (density of 10.49 g/cm^3^ [53]) the contribution to the composite’s weight was counteracted by its high electrical conductivity. Consequently, the material preserved the application potential for the circumstances wherein the weight is of the essence, but high electrical conductivity is required as well.

**Figure 5:**
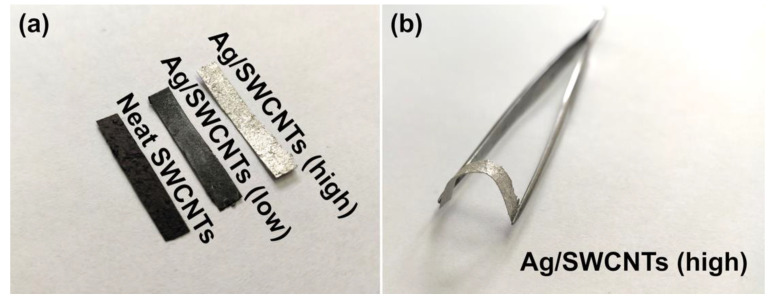
(**a**) The visual appearance of the materials: neat SWCNT film, lightly-doped with Ag, heavily-doped with Ag. (**b**) Demonstration of the flexibility of the heavily-doped SWCNT film.

Visual inspection of the composites also shows that despite the Ag addition, the SWCNT networks preserve their mechanical integrity (Figure 5). Furthermore, with the increase in Ag content, the material becomes grey and eventually silvery as expected. It is also evident that even at the high content of Ag, the material remains highly flexible, as illustrated in Figure 5b.

**Figure 6:**
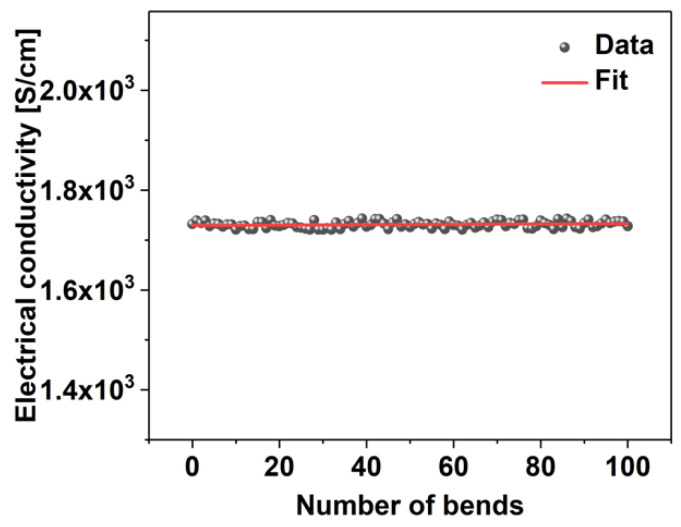
The impact of repeated bending of an SWCNT film heavily-doped with Ag on the electrical conductivity.

Lastly, to ensure the durability of the proposed materials, we inspected the impact of repeated bending of the composite on its electrical conductivity (Figure 6).

For this experiment, the heavily-loaded SWCNTs were employed. The sample was subjected to one hundred bends, during which the electrical conductivity of the network was measured. The results showed that there is virtually no change in the material’s electrical conductivity and the slight variation in this parameter remains within the level of measurement uncertainty. This finding validates that the developed technique of manufacturing metal-nanocarbon composites is promising as it gives robust nanocomposites. Regarding the stability of the values of electrical conductivity in time, when kept in a desiccator, no statistically signifant changes could be observed after 3 months of storage.

## 4. Conclusions

In summary, we demonstrated how Ag-SWCNT composites can be made straightforwardly. The incorporation of Ag during the formation of the SWCNT films enabled its homogeneous distribution in the network. Consequently, a substantial improvement in electrical conductivity was observed. While the addition of a low amount of Ag doubled the electrical conductivity of the material, a boost of nearly 600% was witnessed when a high amount of Ag was employed instead. Interestingly, despite the possible weight burden of Ag, which has a considerable density, the specific electrical conductivity of the material remained at a similar level before and after doping. It appears that the increase in weight was accompanied by sufficiently high electrical conductivity enhancement of the SWCNT ensemble. What is crucial, despite the high content of Ag, the flexibility of the SWCNT network was preserved so that the material could find its use in flexible electronics.

Due to their lightweight, the produced highly conductive nanocomposites are attractive for a spectrum of applications. In particular, the aerospace and automotive industries could significantly gain from their implementation, as the developed materials have encouraging values of specific electrical conductivity. It is clear that more insight is required on how other types of metals would improve the characteristics of nanocarbon networks analogously. For instance, copper of aluminum are lighter than silver, but they offer high conductivity as well. Therefore, it would be interesting to try them as fillers for the SWCNT matrix using the methodology described above. However, for that to happen, appropriate corresponding conductive paints would need to be developed first to adapt and extend the strategy proposed in this paper.

## Data Availability

Data regarding this article is available from the corresponding author upon a reasonable request.

## Supplementary Materials

The following are available online at https://www.mdpi.com/article/10.3390/ma14081956/s1. The Supplementary Material provided contain the chemical analysis of Ag-doped SWCNT films by EDX (Figure S1: The ratio of C (equivalent to the amount of SWCNTs) to Ag measured by EDX).
