# Supplementary material for: A Straightforward Approach to Create Ag/SWCNT Composites
Source: Materials (Basel). 2021 Apr 14;14(8):1956. doi: 10.3390/ma14081956 (PMC8070709; doi:10.3390/ma14081956)
Supplement: Supplementary file 1 [file materials-14-01956-s001.pdf]

materials

MDPI

Supplementary

A Straightforward Approach to Create Ag/SWCNT Composites

Monika Rdest [1], and Dawid Janas [2,*]

[1] Department of Materials Science and Metallurgy, University of Cambridge, 27 Charles Babbage Rd, CB3 0FS Cambridge, UK; Monika.Rdest@gmail.com

[2] Department of Organic Chemistry, Bioorganic Chemistry and Biotechnology, Silesian University of Technology, B. Krzywoustego 4, 44-100 Gliwice, Poland; Dawid.Janas@polsl.pl

* Correspondence: Dawid.Janas@polsl.pl; Tel.: +48-32-237-10-82 (D.J.)

1. EDX analysis

The chemical analysis conducted by EDX overall followed the findings from simple weighing the samples after doping. The Ag-SWCNTs (low) and Ag-SWCNTs (high) composites revealed to have 23 and 61% of Ag by weight, respectively. These values are slightly elevated and translate to Ag:SWCNT ratios of 1:3.33 and 1.57:1, respectively. The discrepancy can be related to the inability of the EDX technique to precisely quantify lighter elements such as carbon. That is because the x-ray yields for such elements are lower than that of heavy elements.

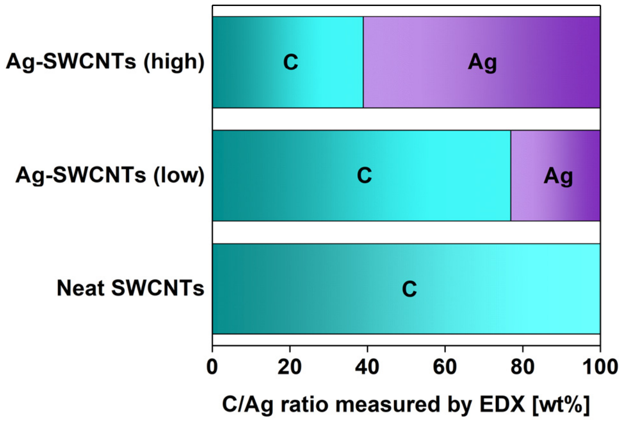

Figure S1. The ratio of C (equivalent to the amount of SWCNTs) to Ag measured by EDX.
